# Predictors of in‐school and out‐of‐school sport injury prevention: A test of the trans‐contextual model

**DOI:** 10.1111/sms.13826

**Published:** 2020-09-26

**Authors:** Alfred S. Y. Lee, Martyn Standage, Martin S. Hagger, Derwin K. C. Chan

**Affiliations:** ^1^ School of Public Health The University of Hong Kong Hong Kong China; ^2^ Centre for Motivation and Health Behaviour Change Department for Health University of Bath Bath UK; ^3^ SHARPP Lab Psychological Sciences University of California Merced CA USA; ^4^ Faculty of Sport and Health Sciences University of University of Jyväskylä Jyväskylä Finland; ^5^ Faculty of Education and Human Development The Education University of Hong Kong Hong Kong China; ^6^ School of Psychology Curtin University Perth WA Australia

**Keywords:** physical education, secondary school sport injury, self‐determination theory, the trans‐contextual model, theory of planned behavior

## Abstract

The current study aimed to predict secondary school students’ motivation toward sport injury prevention in “in‐school” and “out‐of‐school” contexts, and their sport injury prevention behavior at 3‐month follow‐up using the trans‐contextual model (TCM). Hong Kong secondary school students (N = 1566; mean age = 13.34 years, range = 11 to 19; female = 49.42%) were recruited. Participants were asked to complete a survey comprising previously validated scales measuring TCM constructs at baseline and a measure of sport injury prevention behavior at follow‐up three months later. Structural equation modeling (SEM) was used to examine the hypothesized paths among TCM constructs. A SEM specifying hypothesized paths among TCM variables showed acceptable fit with the data (χ^2^(29) = 418.55, CFI = .93, TLI = .90, and RMSEA = .09, 90% CI [.09, .10], and SRMR = .05). Findings supported tenets of the TCM: the effects of perceived autonomy support from PE teachers on in‐school autonomous motivation toward injury prevention, the trans‐contextual relationship between students' “in‐school” and “out‐of‐school” autonomous motivation toward injury prevention, and the effects of autonomous motivation toward injury prevention on social cognitive variables and subsequent sport injury prevention behaviors. Results supported the tenets proposed within the TCM in predicting students' “in‐school” and “out‐of‐school” autonomous motivation toward sport injury prevention. Findings underscore the potential importance of autonomy support from PE teachers in facilitating students’ sport injury prevention behaviors. Further longitudinal and intervention research is warranted to establish temporal and causal effects of TCM variables in sport injury prevention.

## 1 INTRODUCTION

Participation in sport is beneficial to the physical and psychological development of young people^1^, ^2^; yet, it can also pose increased risk of sport injuries.^3^, ^4^ Sport injury can be defined as “any unintentional or intentional damage to the body resulting from participation in sport.”^5^ Sport injuries in young people present a substantive burden in terms of medical costs,^6^, ^7^ time lost in school and sport,^3^, ^8^ and lower future commitment to sport.^9^ The causes of sport injuries are multifactorial.^10^, ^11^ Determinants of sport injury include training load, sex, age, environmental influences, and psychological and physiological conditions.^10^ Research has focused on minimizing sport injury risk, with one highly effective means is the adoption of sport injury prevention behaviors. Sport injury prevention behavior includes, but is not limited to, warm‐up before and cool down after sport,^12^, ^13^ resistance training,^14^ neuromuscular training,^7^ practicing correct landing technique,^15^ and correct application of protective equipment.^16^ However, the prevalence of sport injury among young people is notable. For example, Sheu, Chen, Hedegaard ^17^ found injury rates to be particularly high among children and young people aged between 5 and 14 years (ie, 76.6 sport injuries episodes per 1,000 individuals). It is therefore not surprising that sport injury is considered to be one of the most common injuries among young people.^4^, ^18^

It is well documented that sport injury among young people is more common in an “out‐of‐school” context than in an “in‐school” context (eg, physical education (PE) lessons or other school‐based physical activities).^4^, ^19^ One reason for this is that young people may be more likely to engage in sport injury prevention behaviors when they participate in sport under the supervision of PE teachers than they participate in sport in an “out‐of‐school” context where their adherence to sport injury prevention is more dependent on self‐regulation.^19^, ^20^ Of course, in some cases, out‐of‐school sport participation may be under the auspices of a coach or other supervisor, who may also provide advice and instruction on sport injury prevention behaviors. However, such supervision is not likely to be as pervasive and universal relative to “in‐school” sport participation where the PE teacher is normally present.^20^ For instance, according to the Education Bureau in Hong Kong, sport injury prevention education is a central aim of PE lessons.^21^ Students are also more exposed to sport injury when they are doing sport in recreational contexts compared to school, or when they attend supervised coached sport clubs or organized training outside of school.^4^

In order to gain a better understanding of this phenomenon, it is important to identify the factors that determine why young people are more likely to adopt sport injury prevention strategies in an “in‐school” context compared to “out‐of‐school” contexts.^4^ When performing sport in out‐of‐school contexts, particularly when not supervised, students' participation in injury prevention behaviors is likely to be highly dependent on their personal motivation to do so.^19^ So it is important not only to explore the motivational determinants of sport injury prevention in an “in‐school” context,^20^ but also the motivation to engage in these behaviors outside of school. PE is likely to have a pervasive influence on sport behavior outside of school,^22^, ^23^ so it is also important to establish the extent to which young people perceive their PE teachers are supportive of their sport injury prevention behaviors both inside and outside of school.^7^ To address these proposed areas of research, this study aims to apply the trans‐contextual model,^24^, ^25^ a multi‐theory model of motivation, to identify the determinants of students' motivation toward performing sport injury prevention in “in‐school” and “out‐of‐school” contexts and the processes involved.

The trans‐contextual model of motivation TCM ^24^, ^25^ was initially developed to examine motivation toward physical activities in PE and leisure‐time contexts. The model integrates constructs and hypotheses from self‐determination theory (SDT),^26^ the hierarchical model of intrinsic and extrinsic motivation (HMIEM),^27^ and the theory of planned behavior (TPB).^28^ The principal aim was to explain the processes by which students' motivation in PE classes (ie, in an “in‐school” context) relates to their motivation in leisure‐time physical activity (ie, in an “out‐of‐school” context), and how perceived autonomy support from PE teachers facilitates the transfer of motivation between the contexts. The TCM has three major propositions: (i) the role of perceived autonomy support in determining motivation toward activities in school; (ii) the trans‐contextual relationship between motivation toward activities performed in in‐school and out‐of‐school contexts; and (iii) the role of social cognition constructs as mediators of motivational constructs on actual out‐of‐school physical activity.^25^

### 1.1 Role of perceived autonomy support

The first proposition of the TCM is derived from SDT.^26^ SDT^26^ makes the key distinction between different forms or *qualities* of motivation, namely autonomous and controlled motivation.^26^, ^29^ Autonomous motivation refers to the volitional engagement of activities for self‐endorsed reasons such as out of interest or value. There are three forms of autonomous motivation: intrinsic (performing activities out of interest and enjoyment), integrated (performing activities that represent of one's true sense of self), and identified (performing activities for value or importance attached to its outcome). On the other hand, controlled motivation refers to the engagement in activities for reasons perceived as emanating outside the self, such as internal pressures or external contingencies. Two forms of controlled motivation are proposed: introjected (eg, performing activities for the promotion of ego or for the avoidance of shame) and external (eg, performing activities for obtaining a reward or avoiding punishment). Students who are autonomously motivated toward sport injury prevention behaviors would tend to perform them because they are valued and important to their health and continued sport participation. In contrast, controlled motivated students perform these behaviors because they are required to do so (eg, school regulations, PE teachers’ command), but do not necessarily view them as personally important or useful. Within SDT, autonomous motivation is considered to be high in quality and more adaptive than controlled motivation in that it facilitates behavioral persistence,^25^, ^30^ optimal functioning,^31^ and positive affect.^29^ In contrast, controlled motivation is viewed as being lower in quality and less likely to foster long‐term behavioral commitment because behavior is contingent on factors that lie outside the individual to be sustained, so persistence is unlikely if these factors are not present or are removed.^30^ This does not mean that controlled motivation does not lead to action and persistence. In some contexts, controlled motivation has been shown to be a positive predictor of intention and behavior.^32^, ^33^ However, individuals experienced actions as controlled motivated are only likely to persist as long as the controlling contingencies are present, and when they cease behavior is likely to desist. As a consequence, autonomous motivation is more likely to lead to long‐term behavioral maintenance. Researchers have therefore investigated the conditions which foster autonomous motivation.^25^, ^31^

According to SDT,^26^ contextual factors can facilitate the development of autonomous motivation, particularly through the behaviors of social agents in leadership roles (eg, teachers, managers, coaches). Such social agents can display behaviors and utilize language that supports the autonomy of those acting in those contexts. Autonomy‐supportive behaviors displayed by these agents include providing options and rationales, encouragement, supporting competence, active listening, and showing care and acceptance.^23^, ^29^, ^30^, ^34^ Previous studies that have tested tenets within the TCM have shown that students who perceive their PE teachers as autonomy supportive not only endorse higher autonomous motivation in PE, but also report higher autonomous motivation toward, and intentions to perform, leisure‐time physical activity.^22^, ^23^, ^35^, ^36^ Based on this premise, in the current study we sought to apply the TCM to examine whether autonomy support from PE teachers was related to students’ autonomous motivation toward sport injury prevention in an “in‐school” context and in an “out‐of‐school” context. In past work to apply the TCM to injury prevention, research has only focused on social agents' (eg, coaches and supervisors) generalized autonomy support, and not on specific behaviors that promote injury prevention.^37^, ^38^ In the current study, we aimed to address this evidence gap by examining the effects of students' perception of their PE teachers' support for their autonomy toward sport injury prevention on subsequent in‐school and out‐of‐school motivation toward sport injury prevention.

### 1.2 Transfer of motivation

The second proposition of the TCM was drawn from a central premise within SDT ^26^ and the HMIEM.^27^ According to the HMIEM, the types of motivation that individuals adopt in one context (eg, sport injury prevention in an in‐school context) are transferable to other related contexts (eg, sport injury prevention in an out‐of‐school context). The mechanism behind the trans‐contextual effect is that individuals may develop motivational scripts or schemas in one context, and the scripts are activated when cues related to that behavior are presented in another, albeit similar, context.^24^, ^27^, ^37^, ^39^ Therefore, autonomous and controlled motivation in one context is predicted to be associated with corresponding forms of motivation in different, yet closely related, contexts. Studies have applied this primary TCM tenet to explain motivational transfer in injury prevention and rehabilitation contexts, but past work has focused on elite athletes' or specialist employees' (police officers) injury prevention.^37^, ^38^, ^40^ No previous empirical work has examined this tenet in sport injury prevention among PE students. It is therefore highly worthwhile investigating whether the types of motivation PE students' adopt toward sport injury prevention in an “in‐school” context correspond to the forms of motivation toward sport injury prevention in an “out‐of‐school” context.

### 1.3 Role of social cognition

The third proposition of the TCM is derived from predictions within SDT ^26^ and the TPB.^28^ It is posited that effects of motivational constructs from SDT on future performance of a target behavior will be mediated by the social cognition constructs from the TPB.^28^ Specifically, attitude (ie, positive or negative evaluation of a future target behavior), subjective norms (ie, perceptions of the influence of significant others on the future behavior), and perceived behavioral control (PBC; perceived capability and controllability with respect to the future behavior) are proposed to mediate the relationship between forms of motivation from SDT and intention toward, and participation in, the target behavior. This motivational sequence outlines the process by which motives from SDT lead to future behavior enactment. Consistent with previous research, individuals tend to strategically align their system of beliefs with their reasons or motives so as to enable future pursuit of those behaviors.^41^ Equally congruent with TCM predictions, research has consistently supported links between motives from SDT and beliefs from TPB.^22^, ^23^, ^42^

With respect to sport injury prevention, previous studies have shown that (1) autonomous motivation positively predicts the social cognition constructs from the TPB; (2) the TPB constructs positively predict intention; and (3) intentions positively predict behavior.^33^, ^43^ In addition, research on injury management, including sport injury rehabilitation ^33^, ^44^ and occupational injury prevention and rehabilitation,^37^ has also reported positive effects of autonomous motivation on intention and behavior. Yet, these studies did not examine TCM effects for sport injury among PE students. In the current study, we propose that PE students who are autonomously motivated toward sport injury prevention in an out‐of‐school context are more likely to report higher attitude, subjective norm, PBC, and intention with respect to the corresponding behavior and that these constructs would mediate effects of autonomous motivation on sport injury prevention behavior.

### 1.4 Summary of TCM research and its application in sport injury prevention

There is growing support for the premises of the TCM in behavioral contexts and populations including physical education,^23^ math education,^45^ university education,^46^ sport injury rehabilitation,^40^ injury prevention and rehabilitation in elite athletes ^38^ and police officers,^37^ and avoidance of unintentional doping.^47^ These studies show the TCM to provide a useful model for the processes underpinning participation in health behaviors across contexts. Despite this support, no research has utilized the TCM to explain the motivational determinants PE students' sport injury prevention behavior across “in‐school” and “out‐of‐school” contexts and the processes involved. Further, previous applications of the TCM in injury prevention contexts have predominantly used cross‐sectional designs.^33^, ^37^, ^38^ There is a need to apply the TCM to predict future, subsequent sport injury prevention behaviors. Thus, in the present work we used a prospective study design to address this knowledge gap.

### 1.5 The present study

**FIGURE 1:**
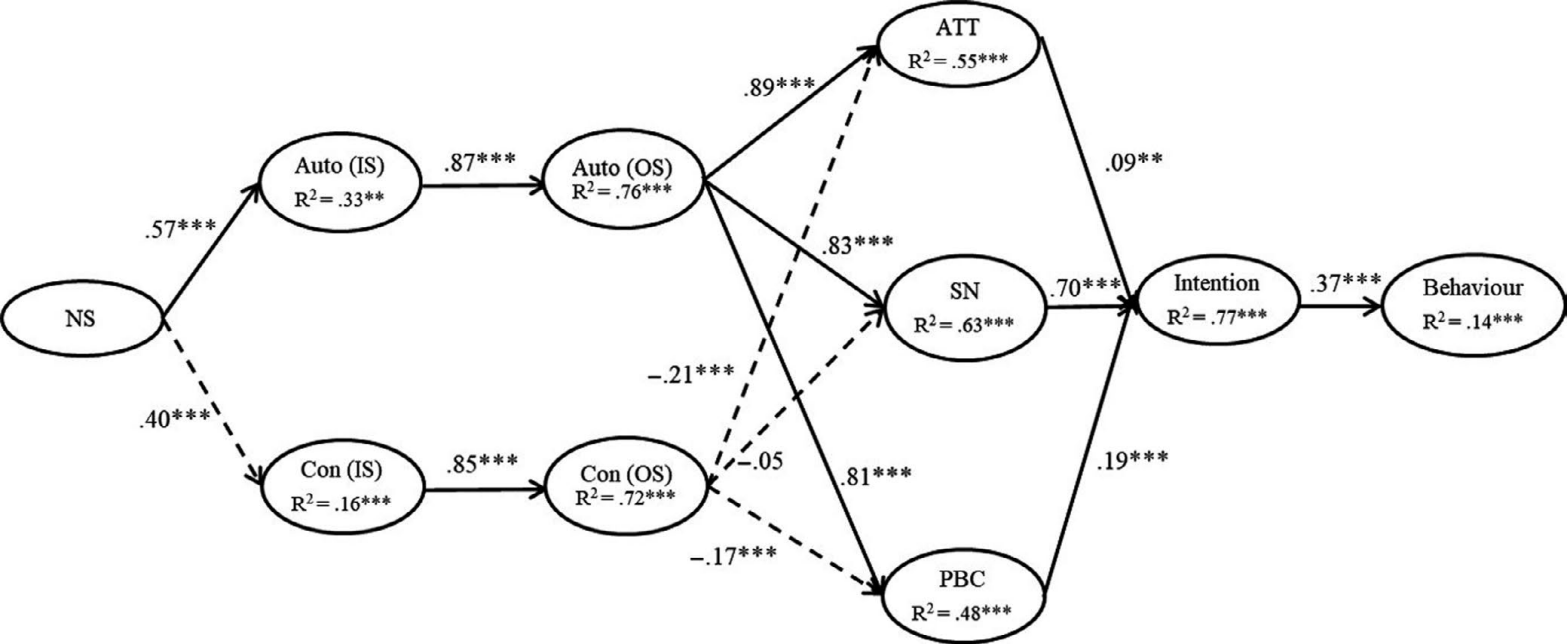
Trans‐contextual model for sport injury prevention. Path estimates with solid lines were hypothesized to be significant and dotted lines were hypothesized to be non‐significant. AS, perceived autonomy support; ATT, attitude; IS, in‐school context; OS, out‐of‐school context; PBC, perceived behavioral control; SN, subjective norms. Behavior = subsequent sport injury prevention behaviors. *P < .05; **P < .01; ***P < .001

In this study, we examined the tenets of the TCM in a sport injury prevention context among PE students using a 3‐month prospective design. Our study included a follow‐up measure of behavior at 3 months which allowed us to conduct a preliminary test of the longer‐term effects of the TCM variables at baseline on sport injury prevention behavior at 3 months later. Based on the propositions of the TCM,^22^, ^25^ and previous findings applying the model in a sport injury prevention context, we proposed the following five sets of hypotheses: a relationship between perceived autonomy support from PE teachers for sport injury prevention behaviors and types of motivation from SDT toward sport injury prevention behaviors “in‐school” (H1); relationships between these motivation types in the two related contexts, “in‐school” and “out‐of‐school” (H2); relationships between SDT motivation types and social cognition constructs from the TPB in an “out‐of‐school context” (H3); relationships between the TPB social cognition constructs, intention, and subsequent sport injury prevention behaviors (H4); and indirect (mediated) effects of perceived autonomy support, SDT motivation types, TPB social cognition constructs, and intentions on subsequent sport injury prevention behaviors, based on TCM predictions (H5). These hypotheses are summarized in Appendix A (Supporting Information) , and the proposed model is presented in Figure 1. Specific hypotheses from each set are as follows:

(H1) Perceived autonomy support from PE teachers would be positively related to in‐school autonomous motivation (H1a), but relations between perceived autonomy support and in‐school controlled motivation (H1b) would be non‐significant.
(H2) Autonomous motivation (H2a) and controlled motivation (H2b) in an in‐school context would be positively related to their corresponding types of motivation in an out‐of‐school context.
(H3) Attitude (H3a^ATT^), subjective norm (H3a^SN^), and PBC (H3a^PBC^) in an out‐of‐school context would be positively related to out‐of‐school autonomous motivation, but these constructs would not be related to out‐of‐school controlled motivation (H3b^ATT^, H3b^SN^, H3b^PBC^).
(H4) Attitude (H4a), subjective norm (H4b), and PBC (H4c) in an out‐of‐school context would be positively related to intention. Intention (H4d) would be positively related to subsequent sport injury prevention behaviors.
(H5) Autonomy support would have positive indirect effects on out‐of‐school autonomous motivation (H5a), intention (H5b), and subsequent sport injury prevention behaviors (H5c). In‐school autonomous motivation would have positive indirect effects on intention (H5d) and subsequent sport injury prevention behaviors (H5e), so would out‐of‐school motivation (H5f, H5g). These effects would be mediated by the constructs that make up the respective motivational sequences of TCM.

## 2 METHOD

### 2.1 Participants

We sent invitations to 462 secondary schools in Hong Kong asking them to participate in a study on sport injury prevention. Six schools agreed to participate. Participants were junior secondary school students (N = 1566; *M* age = 13.34, SD = 1.13; age range = 11 to 19 years; female = 49.42%) in Forms 1 to 3 (equivalent to US 7th to 9th grade). Participants attended two compulsory PE lessons each lasting 35‐40 minutes per week. Participants were prompted report their frequency of sport injuries experienced in the past six months, although they were not provided with a formal definition of sport injury,^48^ and also report how often they were unable to participate in sport outside school, to participate in school PE, and to perform daily activities due to their injuries. On average, participants spent 225.56 minutes per week in sports outside of school (SD = 241.94). Participants reported experiencing an average of 1.28 (SD = 3.20) sport injuries in the last six months. Due to their injuries, students could not participate in sport for an average of 2.08 days (SD = 7.50), were unable to participate in PE for an average of 0.24 days (SD = 1.84), and were unable to carry out daily activities for an average of 1.03 days (SD = 6.19).

### 2.2 Procedures

The study was approved by the Human Research Ethics Committee from the first author's institute (ethics approval number EA1604014). Informed consent forms were signed by both participants and their parents/guardians. Participants were asked to complete the main study survey at baseline and then complete the follow‐up survey three months later. Measures of the TCM variables, participants’ demographic information, and history of sport injury were included in the baseline survey. Self‐reported sport injury prevention behavior in an out‐of‐school context was measured in the follow‐up survey. All survey items have been translated to Chinese and used in previous studies on secondary school students, with satisfactory internal consistency statistics.^22^, ^37^, ^38^, ^42^, ^49^ Full survey measures including item wording, response scales, and previous reliability statistics are available in Appendix B (Supporting Information).

### 2.3 Measures

#### 2.3.1 Perceived autonomy support

Perceived autonomy support from PE teachers was assessed using the six‐item Chinese translation of the sport injury prevention version of the Health Care Climate Questionnaire.^37^, ^38^, ^50^, ^51^ Participant responses were provided on seven‐point scales (1 = *not at all true* and 7 = *very true*).

#### 2.3.2 Sport injury prevention motivation

Forms of motivation from SDT for sport injury prevention in the in‐school context and out‐of‐school context was measured using the 12‐item Chinese version of the Treatment Self‐Regulation Questionnaire TSRQ^52^ adapted for sport injury, the scale that measures individuals' autonomous motivation (6 items) and controlled forms of motivation (6 items) for health behaviors. Participant responses were provided on seven‐point scales (1 = *not at all true* and 7 = *very true*).

#### 2.3.3 Social cognition variables and intention

Attitude (6 items), subjective norms (3 items), PBC (5 items), and intention (3 items) from TPB were measured using Chinese versions of these scales developed in previous research^33^, ^37^, ^42^ and according to published guidelines^53^ and making reference to sport injury prevention behaviors. Participant responses were provided on seven‐point scales (1 = *Strongly disagree* and 7 = *Strongly agree*).

#### 2.3.4 Subsequent sport injury prevention behaviors

Sport injury prevention behavior at 3‐month follow‐up was measured using the Chinese version of the Self‐Reported Injury Prevention Adherence Scale.^38^ The scale consists of 8 items measuring participants' effort (4 items) and frequency (4 items) of preventing sport injury (eg, achieving sport safety, seeking safety advice from others, and avoiding re‐injury). Participant responses were provided on seven‐point scales (1 = *minimum effort/never* and 7 = *maximum effort/very often*).

### 2.4 Data analysis

We calculated descriptive statistics, zero‐order correlations among the TCM constructs, and reliability coefficients for the scales using McDonald's omega.^54^ Single‐indicator structural equation modeling SEM ^55^, ^56^ was performed to examine goodness‐of‐fit and parameter estimates of the TCM using Mplus version 7.2.^57^ We adopted a single‐indicator SEM approach because our TCM model was complex and involved a large number of multi‐item scales in which led to convergence issues when using full‐latent variable SEM analysis with multiple‐indicator factors. Simulation studies suggest that single‐indicator SEM resolves convergence issues and produces reliable model parameter estimates similar to full‐latent variable SEM.^55^ The variables' indicators were generated by taking the average score of the items within the variables and accounting for the error variance using the following formula: (1‐reliability) * sample variance.^56^ Conventional fit indices were used to assess model fit: the comparative fit index (CFI), Tucker‐Lewis index (TLI), root mean square error of approximation (RMSEA), and standardized root mean square residual SRMR.^58^ Traditional cutoff criteria of CFI and TLI (ie, >0.90), and RMSEA and SRMR (ie, <0.08) were applied to indicate acceptable fit. For the mediation analyses, indirect effects of the predicted paths were examined.

Regarding treatment of missing data, 122 out of 1566 (7.79%) participants did not complete the 3‐month follow‐up survey due to withdrawal of consent and loss to follow‐up. We adopted the maximum‐likelihood robust estimation with robust standard errors for the SEM, which adjusts the likelihood function so that each case contributes information on the observed variables. Studies have supported the adequacy of the maximum‐likelihood estimation in handling missing data patterns. Data files, analysis scripts, and outputs for the present study are available online: https://osf.io/c7pqm/.

## 3 RESULTS

### 3.1 Preliminary analysis

Descriptive statistics and intercorrelations for the study variables are presented in Appendix C (Supporting Information). The scores of the study variables exhibited acceptable internal consistency (ω range = .83 to .94).

### 3.2 Structural equation models

The proposed model showed acceptable fit with the data, χ^2^(29) = 418.55, CFI = .93, TLI = .90, and RMSEA = .09, 90% CI [.09, .10], and SRMR = .05. The standardized parameter estimates among model constructs are presented in Figure 1.

#### 3.2.1 Direct effects

We found positive, statistically significant effects of perceived autonomy support from the PE teacher on students' “in‐school” autonomous motivation (H1a, β = .57, *P* < .01). Contrary to hypotheses, we also found a positive, statistically significant effect of perceived autonomy support on “in‐school” controlled motivation (H1b, β = .40, *P* < .01). For the trans‐contextual effects, there were positive, statistically significant effects of “in‐school” autonomous and controlled forms of motivation on their corresponding “out‐of‐school” forms (H2a, autonomous motivation: β = .87, *P* < .01; H2b, controlled motivation: β = .85, *P* < .01), consistent with the hypotheses. As predicted, we also found positive, statistically significant effects of “out‐of‐school” autonomous motivation on attitude (H3a^ATT^, β = .89, *P* < .01), subjective norms (H3a^SN^, β = .83, *P* < .01), and PBC (H3a^PBC^, β = .81, *P* < .01). There were negative, statistically significant effects of the social cognitive constructs on “out‐of‐school” controlled motivation (H3b^ATT^, H3b^PBC^, β = −.21 to −.17, *P* < .01), leading us to reject these hypotheses. The only exception was the effect of controlled motivation on subjective norms, which was not statistically significant (H3b^SN^ β = −.05, *P* > .05). There were positive, statistically significant effects of attitude (H4a, β = .09, *P* < .01), subjective norms (H4b, β = .70, *P* < .01), and PBC (H4c, β = .19, *P* < .01) on intention, as predicted. Finally, we found a positive, statistically significant effect of intention on sport injury prevention behavior (H4d, β = .37, *P* < .01). In our supplementary analyses, we controlled the effects of age and gender in the TCM. However, there were very few effects of these demographic constructs. Inclusion of these constructs also meant that the model fit indices dropped slightly. We decided to omit age and gender from the model for a more parsimonious model. The results are available in Appendix D (Supporting Information).

#### 3.2.2 Indirect effects

Parameter estimates for indirect effects are summarized in Appendix E (Supporting Information). We found a positive, statistically significant indirect effect of perceived autonomy support on “out‐of‐school” autonomous motivation (H5a, β = .50, *P* < .01) mediated by “in‐school” autonomous motivation. There were also a positive, statistically significant indirect effects of perceived autonomy support on intention (H5b, β = .38, *P* < .01) and behavior (H5c, β = .14, *P* < .01) mediated by “in‐school” autonomous motivation, “out‐of‐school” autonomous motivation, the TPB constructs and, in the case of behavior, intention. There were also statistically significant positive indirect effects of “in‐school” autonomous motivation and “out‐of‐school” autonomous motivation on intention and behavior (H5d‐H5g, β = .26‐.82, *P* < .01) mediated by the TCM constructs.

## 4 DISCUSSION

We applied the TCM to identify the determinants of school students’ “in‐school” and “out‐of‐school” motivation toward sport injury prevention behavior and describe the processes involved. A prospective survey design was used to test the proposed effects of perceived autonomy support and forms of motivation in a PE context on forms of motivation, social cognition constructs, intentions, and injury prevention behavior in an out‐of‐school context. Results supported our five main hypotheses. Specifically, we found positive effects for perceived autonomy support from PE teachers on forms of motivation from SDT in an in‐school context; effects of motivation types in an “in‐school” context on their respective types in an “out‐of‐school” context; effects of motivation types on social cognition constructs from the TPB in an “out‐of‐school” context; effects of social cognition constructs on intention and sport injury prevention behavior; and indirect effects of perceived autonomy support and forms of autonomous motivation on intentions and behavior consistent with the TCM. Collectively, the findings of the current study extend the application of the TCM in a different behavioral context and to the prediction of subsequent sport injury prevention behaviors.

### 4.1 Perceived autonomy support

Consistent with SDT,^29^ the TCM,^25^ and past empirical research,^22^, ^46^ perceived autonomy support from PE teachers was a significant predictor of students' autonomous motivation toward sport injury prevention. However, perceptions of autonomy support were also associated with controlled motivation, although the effect was much smaller. The latter finding for controlled motivation is not consistent with the predictions of SDT^29^ and the TCM.^23^ Theoretically, individuals tend to endorse controlled motivation when the environment does not support autonomy, such as when teachers display controlling behaviors and use controlling language.^59^ Nonetheless, this is not the first study to report autonomy support to be positively associated with controlled motivation.^46^ For example, Chan et alChan, Yang, Hamamura, Sultan, Xing, Chatzisarantis, Hagger^46^ reported that perceived autonomy support from lecturers was positively associated with controlled motivation toward “in‐lecture” learning in multiple samples. One possible explanation for this relationship may be that controlled motivation captures forms of motivation that sit at intermediate positions on the continuum of internalization from autonomous to controlled.^52^ In fact, there is research demonstrating positive relations between autonomous forms of motivation such as identification and more controlled forms such as introjection. Yet, the size of the effect of perceived autonomy support on controlled motivation was smaller than the effect on autonomous motivation, which is consistent with the prediction that perceived autonomy support effectively captures teachers' autonomy‐supportive teaching styles and less likely captures controlled forms.

### 4.2 Transfer of motivation

The trans‐contextual effects of motivation in our study were closely aligned with the TCM propositions and previous empirical findings.^24^, ^40^, ^60^ Specifically, we found trans‐contextual effects of the “in‐school” autonomous and controlled motivation, on their corresponding “out‐of‐school” form of motivation. Such findings indicate that students endorsing particular types of motivation toward sport injury prevention “in‐school” tended to do so “out‐of‐school.” The size of the trans‐contextual effects were also consistent with those reported previously.^46^ Within the context of sport injury behavior, the present findings suggest that both forms of motivation tend to translate across contexts, which is in keeping with the proposed contextual interplay between forms of motivation as proposed in the HMIEM and TCM.

### 4.3 Motivation and social cognition

Consistent with predictions within the TCM and aligned with previous research in multiple contexts,^25^ including sport injury prevention,^46^, ^47^ current findings supported relations between autonomous motivation toward sport injury prevention in PE and outside of school on beliefs from the social cognition constructs from the TPB and, indirectly, on intentions and behavior in a sport injury prevention context. This is consistent with the theoretical prediction that students with autonomous motives toward injury prevention tend to bring their beliefs and intentions into line with those motives, a strategic process that enables them to pursue those behaviors in future. In addition, controlled motivation was negatively related to attitude and PBC. The findings in previous research have shown relatively inconsistent effects for this relationship, with some showing negative effects,^32^ some showing positive effects,^46^ and others showing no effects.^44^ However, the current findings are in line with the TCM propositions, which suggest that individuals tend to align their system of beliefs with their reasons or motives so as to enable future pursuit of those behaviors. If students engaged in sport injury prevention for controlled motives they would be unlikely to form positive beliefs on sport injury prevention in future “out‐of‐school” contexts because it was likely that the reinforcing contingencies that led them to perform sport injury prevention behaviors are not likely to be present in that alternative context.^41^ Current findings therefore highlight the importance of autonomous motivation in facilitating future sport injury prevention behaviors among PE students.

Although attitudes, subjective norms, and PBC all predicted intentions toward, and participation in, sport injury behavior, it is important to note that subjective norms had the largest effects on intention. Although this runs against previous meta‐analytic research on the TPB across many contexts in which attitudes and perceived behavioral control tended to have stronger effects, it is in line with previous research on sport injury prevention.^33^, ^37^ One possible explanation for the stronger effect of social influences in the current model is the cultural background of our sample.^46^, ^61^ Our participants were all recruited from secondary schools in Hong Kong where people tend to endorse collectivist norms.^61^ Studies have suggested that individuals from national groups in which collectivistic values pervade are more likely to pay greater heed to normative influences when forming intentions relative to individuals from national groups where individualist value predominate.^37^, ^61^, ^62^, ^63^ Replication of this model of sport injury prevention in other national groups with different cultural norms, as well as formally measuring cultural orientations, may shed further light on this speculative explanation.

### 4.4 Prediction of behavior

An important finding of the current model is that perceived autonomy support and in‐school and out‐of‐school autonomous motivation were indirectly related to sport injury prevention intentions and behavior. These results support the hypotheses of the TCM and also meta‐analytic findings applying the model,^25^ but are seldom supported in empirical studies. These findings are important because they highlight the value of the model for behaviour change interventions in sport injury prevention.^64^ Behavioral interventions aimed at promoting sport injury prevention could focus on changing any one of the constructs in the motivational sequence and in either of the two contexts. These data provide evidence to suggest that interventions targeting change in autonomous motivation toward sport injury prevention in PE may be a possible means to promote sport injury prevention behavior outside of school. Promoting autonomous motivation in PE may be achieved through training teachers to be more autonomy supportive toward sport injury prevention in lessons ^37^, ^40^, ^65^ and using a number of SDT behavior change techniques.^66^ However, research would need to confirm whether the model is able to explain change in outcomes as preliminary research seems to suggest,^67^ and whether interventions targeting particular constructs lead to subsequent changes in behavior.

### 4.5 Limitations and future directions

There are a number of limitations of the present research. These data are correlational, so directional and causal effects are inferred from theory alone and not the data. Research is needed to develop interventions targeting change in TCM constructs and examine their effects on other model constructs and sport injury prevention behavior. For example, researchers may look to conduct interventions wherein they seek to train PE teachers to adopt autonomy‐supportive behaviors^66^ and examine the effect of their subsequent teaching on in‐school and out‐of‐school motivation, social cognition constructs, intentions, and sport injury prevention behavior.^68^ The TCM also suggests that interventions could target other model constructs within the motivational sequence, such as attitudes. This might involve persuasive communications aimed at highlight the pros and diminish the cons with respect to sport injury prevention.^64^, ^69^ The current research also relied exclusively on self‐report scales, particularly for the measurement of sport injury prevention behavior. Despite good validity data in support of the sport injury prevention adherence scale,^33^, ^37^, ^38^ social desirability remains a real issue. In addition, the exclusive use of self‐report measures likely introduced common method variance to the relations, which may inflate effects. Researchers should seek to adopt non–self‐report or external measures to assess students' sport injury prevention behavior such as clinically verified data on injury (eg, sport injury rates, severity of the injury), and coaches, peer or parents' evaluation of young people's sport injury prevention behavior.^70^ In the current study, we also did not differentiate between different types of behaviors that students may engage in to prevent injury such as warming up, stretching, and strengthening exercises, so the focus on the study is on a category of behaviors rather than a specific behavior. Future research may seek to examine the TCM effects for specific behaviors of sport injury prevention and investigate how they relate to specific types of injury (eg, soft tissue injuries, impact trauma, overuse injuries). Such research is highly important because the type of sport injury preventive behaviors could vary according to sport type, sport level, time spend in sport and developmental stage of individuals and other personal and environmental factors (eg, environmental hazards, availability of protective kits), and testing the tenets of TCM of sport injury prevention should take these factors into account. In our current study, we have only examined the transfer of motivation from in‐school toward out‐of‐school. We focused on this directional hypothesis for pedagogical reasons. PE students learn sport injury prevention in PE and, with sufficient support and motivation from their teacher, may apply this when performing sports outside of school. However, we also speculated that there may be some cases where the opposite might happen (eg, such as when student athletes receive sport coaching in out‐of‐school context and then perform the same sport in school). Thus, for future research, we would encourage researchers to investigate the reciprocal relationships between in‐school and out‐of‐school motivation.^42^ Finally, given the homogeneity of the sampling methods, it is also important to note that we cannot infer that the current findings will generalize to samples in other national, cultural, or ethnic contexts.^71^ It is therefore highly recommended that the current study be replicated in other countries and cultural or ethnic groups and comparisons made to estimate the extent to which these effects can be generalized.

## 5 PERSPECTIVES

The present study applied the trans‐contextual model (TCM) to sport injury prevention behaviors in secondary school students. Results supported TCM premises: perceived autonomy support in a PE context and forms of motivation from SDT in “in‐school” and “out‐of‐school” contexts were indirectly related to intention, and sport injury prevention behaviors outside of school mediated by social cognition constructs from the TPB. Findings provide preliminary evidence that may inform behavior change interventions aimed at promoting sport injury prevention behaviors by identifying relevant target constructs. Future intervention research is warranted to establish the causal effects of the TCM constructs on behavior in this context.

## Supporting information

Supplementary Material
