# Supplementary material for: Predictors of in‐school and out‐of‐school sport injury prevention: A test of the trans‐contextual model
Source: Scand J Med Sci Sports. 2020 Sep 26;31(1):215–25. doi: 10.1111/sms.13826 (PMC7756760; doi:10.1111/sms.13826)
Supplement: Supplementary file 1 — Supplementary Material [file SMS-31-215-s001.docx]

**Appendix A.**

*Summary of hypothesised direct and indirect effects in the proposed trans-contextual model.*

|  | Independent Variable | Dependent Variable | Mediator(s) | Hypothesis |
| --- | --- | --- | --- | --- |
| H1a | Autonomy Support | Autonomous Motivation (IS) | - | Positive Effect |
| H1b | Autonomy Support | Controlled Motivation (IS) | - | No Effect |
| H2a | Autonomous Motivation (IS) | Autonomous motivation (OS) | - | Positive Effect |
| H2b | Controlled Motivation (IS) | Controlled motivation (OS) | - | Positive Effect |
| H3a^ATT^ | Autonomous Motivation (OS) | Attitude | - | Positive Effect |
| H3a^SN^ | Autonomous Motivation (OS) | Subjective norms | - | Positive Effect |
| H3a^PBC^ | Autonomous Motivation (OS) | PBC | - | Positive Effect |
| H3b^ATT^ | Controlled Motivation (OS) | Attitude | - | No Effect |
| H3b^SN^ | Controlled Motivation (OS) | Subjective norms | - | No Effect |
| H3b^PBC^ | Controlled Motivation (OS) | PBC | - | No Effect |
| H4a | Attitude | Intention | - | Positive Effect |
| H4b | Subjective Norms | Intention | - | Positive Effect |
| H4c | PBC | Intention | - | Positive Effect |
| H4d | Intention | Subsequent Behaviour | - | Positive Effect |
| H5a | Autonomy Support | Autonomous motivation (OS) | Autonomous motivation (IS) | Positive Effect |
| H5b | Autonomy Support | Intention | Autonomous motivation (IS)  Autonomous motivation (OS)  Intention antecedents | Positive Effect |
| H5c | Autonomy Support | Subsequent Behaviour | Autonomous motivation (IS)  Autonomous motivation (OS)  Intention antecedents  Intention | Positive Effect |
| H5d | Autonomous Motivation (IS) | Intention | Autonomous motivation (OS)  Intention antecedents | Positive Effect |
| H5e | Autonomous Motivation (IS) | Subsequent Behaviour | Autonomous motivation (OS)  Intention antecedents  Intention | Positive Effect |
| H5f | Autonomous Motivation (OS) | Intention | Intention antecedents | Positive Effect |
| H5g | Autonomous Motivation (OS) | Subsequent Behaviour | Intention antecedents  Intention | Positive Effect |

*Note*. IS = in-school context; OS = out-of-school context; ATT = attitude; SN = subjective norms; PBC = perceived behavioural control.

**Appendix B..** Details of Measures Used in Trans-Contextual Model for In-School and Out-Of-School Sport (Online Supplemental Material)

| Measure | Subscale (if applicable) | Items | Scale (if applicable) | Previous studies and its reliability |
| --- | --- | --- | --- | --- |
| Perceived autonomy support from PE teachers^[[1]](#footnote-1)^ |  | I feel that my PE teacher has provided me choices and options.  I feel understood by my PE teacher.  My PE teacher conveys confidence in my ability to make changes.  My PE teacher encourages me to ask questions.  My PE teacher listens to how I would like to do things.  My PE teacher tries to understand how I see things before suggesting a new way to do things. | 1 = Strongly agree,  7 = Strongly disagree | Sport injury prevention version:  [Chan, Hagger ^1^](#_ENREF_1)  Age group: M_age_ = 16.79, SD = 2.80  Cronbach’s Alpha: .92  Sport version:  [Adie, Duda, Ntoumanis ^2^](#_ENREF_2)  Age group: M_age_ = 13.82; SD = 1.99  Cronbach’s Alpha: .90 |
| Perceived locus of causality (school) | Autonomous motivation^[[2]](#footnote-2)^ | Stem: In my PE lesson, …  … I want to prevent or avoid sport injury because I feel that I want to take responsibility for my own health.  …I want to prevent or avoid sport injury because I personally believe it is the best thing for my health.  … I want to prevent or avoid sport injury because I have carefully thought about it and believe it is very important for many aspects of my life.  … I want to prevent or avoid sport injury because it is an important choice I really want to make.  … I want to prevent or avoid sport injury because it is consistent with my life goals.  …I want to prevent or avoid sport injury because it is very important for being as healthy as possible. | 1 = Not true at all, 7 = Very true | Sport injury prevention version: [Chan, Zhang, Lee, Hagger ^3^](#_ENREF_3)  Age group: M_age_ = 14.42, SD = 1.75  Cronbach’s Alpha: .82 |
|  | Controlled motivation^[[3]](#footnote-3)^ | …I want to prevent or avoid sport injury because I would feel guilty or ashamed of myself if did not.  … I want to prevent or avoid sport injury because others would be upset with me if I did not.  …I want to prevent or avoid sport injury because I would feel bad about myself if I did not.  … I want to prevent or avoid sport injury because I feel pressure from others to prevent any sport injury that could happen to me.  … I want to prevent or avoid sport injury because I want others to approve of me.  … I want to prevent or avoid sport injury because I want others to see I can do it. |  | Sport injury prevention version: [Chan, Hagger ^4^](#_ENREF_4)  Age group: M_age_ = 16.8, SD = 2.8  Cronbach’s Alpha: .74 |
| Perceived locus of causality (out-of-school) | Autonomous motivation^2^ | Stem: When I participate in sport after-school, …  … I want to prevent or avoid sport injury because I feel that I want to take responsibility for my own health.  …I want to prevent or avoid sport injury because I personally believe it is the best thing for my health.  … I want to prevent or avoid sport injury because I have carefully thought about it and believe it is very important for many aspects of my life.  … I want to prevent or avoid sport injury because it is an important choice I really want to make.  … I want to prevent or avoid sport injury because it is consistent with my life goals.  …I want to prevent or avoid sport injury because it is very important for being as healthy as possible. | 1 = Not true at all, 7 = Very true | Sport injury prevention version: [Chan, Zhang, Lee, Hagger ^3^](#_ENREF_3)  Age group: M_age_ = 14.42, SD = 1.75  Cronbach’s Alpha: .82 |
|  | Controlled motivation^3^ | …I want to prevent or avoid sport injury because I would feel guilty or ashamed of myself if did not.  … I want to prevent or avoid sport injury because others would be upset with me if I did not.  …I want to prevent or avoid sport injury because I would feel bad about myself if I did not.  … I want to prevent or avoid sport injury because I feel pressure from others to prevent any sport injury that could happen to me.  … I want to prevent or avoid sport injury because I want others to approve of me.  … I want to prevent or avoid sport injury because I want others to see I can do it. |  | Sport injury prevention version: [Chan, Hagger ^4^](#_ENREF_4)  Age group: M_age_ = 16.8, SD = 2.8  Cronbach’s Alpha: .74 |
| Theory of planned behavior | Intention^[[4]](#footnote-4)^ | I intend to carry out all required safety procedures to reduce the likelihood or severity of injury in the forthcoming month  I will try to put great effort into following all required safety procedures to reduce the likelihood or severity of injury in the forthcoming month  I plan to follow all required safety procedures to reduce the likelihood or severity of injury in the forthcoming month | 1 = Strongly disagree, 7 = Strongly agree | Sport injury prevention version: [Chan, Hagger ^4^](#_ENREF_4)  Age group: M_age_ = 16.8, SD = 2.8  Cronbach’s Alpha: .80  Physical activities version:  [González‐Cutre, Sicilia, Beas‐Jiménez, Hagger ^5^](#_ENREF_5)  Age group: M_age_ = 13.90, SD = 1.33  Cronbach’s Alpha: .84 |
|  | Attitude^[[5]](#footnote-5)^ | Stem: Following all required safety procedures to reduce the likelihood or severity of injury …  Worthless – Valuable.  Harmful – Beneficial.  Unpleasant – Pleasant.  Bad – Good.  Not virtuous – Virtuous. | Seven-point sematic differential scales | Sport injury prevention version: [Chan, Zhang, Lee, Hagger ^3^](#_ENREF_3)  Age group: M_age_ = 14.42, SD = 1.75  Cronbach’s Alpha: .90 |
|  | Subjective norm^[[6]](#footnote-6)^ | Most people who are important to me think that I should follow all required safety procedures to reduce the likelihood or severity of injury in the forthcoming month.  It is expected of me that I follow all required safety procedures to reduce the likelihood or severity of injury in the forthcoming month.  The people in my life whose opinions I value would approve me to follow all required safety procedures to reduce the likelihood or severity of injury in the forthcoming month. | 1 = Strongly disagree,  7 = Strongly agree | Sport injury prevention version: [Chan, Zhang, Lee, Hagger ^3^](#_ENREF_3)  Age group: M_age_ = 14.42, SD = 1.75  Cronbach’s Alpha: .85 |
|  | Perceived behavioral control^[[7]](#footnote-7)^ | It is possible for me to follow all required safety procedures to reduce the likelihood or severity of injury in the forthcoming month.  If I want to, I could follow all required safety procedures to reduce the likelihood or severity of injury in the forthcoming month.  I have complete control over how to follow all required safety procedures to reduce the likelihood or severity of injury in the forthcoming month.  It is mostly up to me whether or not I follow all required safety procedures to reduce the likelihood or severity of injury in the forthcoming month.  It is easy for me to follow all required safety procedures to reduce the likelihood or severity of injury in the forthcoming month. | 1 = Strongly disagree,  7 = Strongly agree | Sport injury prevention version: [Chan, Zhang, Lee, Hagger ^3^](#_ENREF_3)  Age group: M_age_ = 14.42, SD = 1.75  Cronbach’s Alpha: .89 |
| Sport injury prevention behaviour^[[8]](#footnote-8)^ | Frequency | Stem: When I participate in sport after-school, …  How often do you work on achieving safety objectives when you do sport (e.g., checking sport equipment, inspecting hazards in the training environments, wearing protective kits)?  How often do you work on improving your physical/ mental conditions to avoid injuries (e.g., warm-up, stretching, physical conditioning, resting adequately)?  How often do you work on avoiding re-injury for your old injuries (e.g., use of ice, banding, taking supplements)?  How often do you seek injury prevention/ safety advice from others (e.g., athletes, coaches, and medical staff)? | 1 = Never,  7 = Very often; | Sport injury prevention version:  [Chan, Hagger ^1^](#_ENREF_1)  Age group: M_age_ = 16.79, SD = 2.80  Cronbach’s Alpha: .80 |
|  | Effort | How much effort do you put on achieving safety objectives during your working hours (e.g., checking sport equipment, inspecting hazards in the training environments, wearing protective kits)?  How much effort do you put on improving your physical/ mental conditions to avoid injuries (e.g., warm-up, stretching, resting adequately)?  How much effort do you put on avoiding re-injury for your old injuries (e.g., use of ice, banding, taking supplements)?  How much effort do you put in an attempt to seek injury prevention/ safety advice from others (e.g., athletes, coaches, and medical staff)? | 1 = Minimum effort, 7 = Maximum effort | Sport injury prevention version:  [Chan, Hagger ^1^](#_ENREF_1)  Age group: M_age_ = 16.79, SD = 2.80  Cronbach’s Alpha: .80 |
|  |  |  |  |  |

**Appendix C.**

| Variables | 1 | 2 | 3 | 4 | 5 | 6 | 7 | 8 | 9 | 10 |
| --- | --- | --- | --- | --- | --- | --- | --- | --- | --- | --- |
| 1. Autonomy Support | 1 |  |  |  |  |  |  |  |  |  |
| 2. Autonomous Motivation (IS) | .55*** | 1 |  |  |  |  |  |  |  |  |
| 3. Controlled Motivation (IS) | .39*** | .65*** | 1 |  |  |  |  |  |  |  |
| 4. Autonomous Motivation (OS) | .54*** | .88*** | .57*** | 1 |  |  |  |  |  |  |
| 5. Controlled Motivation (OS) | .39*** | .57*** | .85*** | .75*** | 1 |  |  |  |  |  |
| 6. Attitude | .41*** | .67*** | .34*** | .74*** | .47*** | 1 |  |  |  |  |
| 7. Subjective Norm | .40*** | .64*** | .43*** | .73*** | .56*** | .64*** | 1 |  |  |  |
| 8. PBC | .34*** | .56*** | .32*** | .63*** | .43*** | .61*** | .77*** | 1 |  |  |
| 9. Intention | .42*** | .63*** | .40*** | .69*** | .50*** | .65*** | .85*** | .72*** | 1 |  |
| 10. Subsequent Behaviour | .28*** | .39*** | .25*** | .39*** | .28*** | .32*** | .34*** | .29*** | .36*** | 1 |
| McDonalds' Omega | .94 | .84 | .83 | .87 | .88 | .89 | .82 | .88 | .92 | .91 |
| Cronbach’s Alpha | .94 | .84 | .83 | .87 | .88 | .89 | .82 | .88 | .92 | .91 |
| Mean | 4.60 | 4.94 | 3.86 | 4.81 | 4.13 | 4.99 | 4.62 | 4.74 | 4.55 | 4.08 |
| Standard Deviation | 1.34 | 1.10 | 1.26 | 1.13 | 1.29 | 1.10 | 1.14 | 1.07 | 1.17 | 1.24 |

*Descriptive Statistics and Correlations between Variables (N = 1,566)*

Note. IS = in-school context; OS = out-of-school context; PBC = perceived behavioural control.

*** *p* <.001

**Appendix D.**

*Parameter Estimates of the Trans-Contextual Model for Injury Prevention Controlling for Age and Sex*

| Paths | β [95% CI] |
| --- | --- |
|  |  |
| Proposed TCM Path |  |
| AS → Auto (IS) | .57*** [.53 - .61] |
| AS → Con (IS) | .40*** [.36 - .45] |
| Auto (IS) → Auto (OS) | .87*** [.85 - .90] |
| Con (IS) → Con (OS) | .85*** [.82 - .88] |
| Auto (OS) → Attitude | .88*** [.82 - .93] |
| Auto (OS) → Subjective Norms | .84*** [.77 - .91] |
| Auto (OS) → PBC | .81*** [.74 - .88] |
| Con (OS) → Attitude | -.21*** [-.27 - -.14] |
| Con (OS) → Subjective Norms | -.06 [-.14 - .02] |
| Con (OS) → PBC | -.18*** [-.26 - -.10] |
| Attitude → Intention | .09** [.05 - .14] |
| Subjective Norms → Intention | .70*** [.64 - .76] |
| PBC → Intention | .19*** [.12 - .25] |
| Intention → Prospective Behaviour | .37*** [.32 - .42] |
|  |  |
| Covariates |  |
| Age → Auto (IS) | -.07** [-.10 - -.02] |
| Age → Con (IS) | .09** [.04 - .13] |
| Age → Auto (OS) | .01 [-.02 - .04] |
| Age → Con (OS) | -.03 [-.06 - -.00] |
| Age → Attitude | -.06** [-.09 - -.02] |
| Age → Subjective Norms | .02 [-.02 - .05] |
| Age → PBC | .01 [-.03 - .05] |
| Age → Intention | -.01 [-.04 - .02] |
| Age → Prospective Behaviour | .00 [-.04 - .05] |
| Sex → Auto (IS) | -.00 [-.04 - -.04] |
| Sex → Con (IS) | .03 [-.01 - .07] |
| Sex → Auto (OS) | -.02 [-.05 - -.02] |
| Sex → Con (OS) | .00 [-.03 - .04] |
| Sex → Attitude | .04 [.01 - .08] |
| Sex → Subjective Norms | .04 [.01 - .08] |
| Sex → PBC | .06* [.02 - .10] |
| Sex → Intention | .02 [-.01 - .05] |
| Sex → Prospective Behaviour | -.01 [-.05 - .03] |

*Note*. IS = in-school context; OS = out-of-school context; AS = perceived autonomy support;

Auto = autonomous motivation; Con = Controlled motivation; PBC = Perceived Behavioural Control

* *p* <.05 ** *p* <.01*** *p* <.001

Appendix E.

*Parameter Estimates for Direct, Indirect, and Total Effects from Mediation Analyses of the Proposed Structural Equation Model Based on the Trans-Contextual Model for Injury Prevention*

| Hypotheses | Paths | Mediator (s) | Indirect effects [95% CI] |
| --- | --- | --- | --- |
| H5a | AS → Auto (OS) | Auto (IS) | .50***[.46 - .54] |
| H5b | AS → Intention | Auto (IS),  Auto (OS),  Intention antecedents | .38***[.34 - .41] |
| H5c | AS → Subsequent Behaviour | Auto (IS),  Auto (OS),  Intention antecedents,  Intention | .14***[.12 - .16] |
| H5d | Auto (IS) → Intention | Auto (OS),  Intention antecedents | .71***[.66 - .76] |
| H5e | Auto (IS) → Subsequent Behaviour | Auto (OS),  Intention antecedents,  Intention | .26***[.22 - .30] |
| H5f | Auto (OS) → Intention | Intention antecedents | .82***[.76 - .87] |
| H5g | Auto (OS) → Subsequent Behaviour | Intention antecedents,  Intention | .30***[.26 - .35] |

*Note*. IS = in-school context; OS = out-of-school context; AS = perceived autonomy support; Auto = autonomous motivation.

*** *p* <.001

*Parameter Estimates for Direct, Indirect, and Total Effects from Mediation Analyses of the Proposed Structural Equation Model Based on the Trans-Contextual Model for Injury Prevention*

| Hypotheses | Paths | Mediator (s) | Indirect effects [95% CI] |
| --- | --- | --- | --- |
| H5a | AS → Auto (OS) | Auto (IS) | .50***[.46 - .54] |
| H5b | AS → Intention | Auto (IS),  Auto (OS),  Intention antecedents | .38***[.34 - .41] |
| H5c | AS → Subsequent Behaviour | Auto (IS),  Auto (OS),  Intention antecedents,  Intention | .14***[.12 - .16] |
| H5d | Auto (IS) → Intention | Auto (OS),  Intention antecedents | .71***[.66 - .76] |
| H5e | Auto (IS) → Subsequent Behaviour | Auto (OS),  Intention antecedents,  Intention | .26***[.22 - .30] |
| H5f | Auto (OS) → Intention | Intention antecedents | .82***[.76 - .87] |
| H5g | Auto (OS) → Subsequent Behaviour | Intention antecedents,  Intention | .30***[.26 - .35] |

*Note*. IS = in-school context; OS = out-of-school context; AS = perceived autonomy support; Auto = autonomous motivation.

* *p* <.05 ** *p* <.01*** *p* <.001

**Reference**

1. Chan DKC, Hagger MS. Transcontextual development of motivation in sport injury prevention among elite athletes. *J Sport Exerc Psychol.* 2012;34(5):661-682.

2. Adie JW, Duda JL, Ntoumanis N. Perceived coach-autonomy support, basic need satisfaction and the well-and ill-being of elite youth soccer players: A longitudinal investigation. *Psychol Sport Exerc.* 2012;13(1):51-59.

3. Chan DKC, Zhang L, Lee ASY, Hagger MS. Reciprocal relations between autonomous motivation from self-determination theory and social cognition constructs from the theory of planned behavior: A cross-lagged panel design in sport injury prevention. *Psychol Sport Exerc.* 2020;48:101660.

4. Chan DKC, Hagger MS. Self-determined forms of motivation predict sport injury prevention and rehabilitation intentions. *J Sci Med Sport.* 2012;15(5):398-406.

5. González‐Cutre D, Sicilia A, Beas‐Jiménez M, Hagger M. Broadening the trans‐contextual model of motivation: A study with Spanish adolescents. *Scandinavian journal of medicine & science in sports.* 2014;24(4).

1. Higher scores indicate higher perception of autonomy support from PE teachers. [↑](#footnote-ref-1)
2. Higher scores indicate students are more autonomously motivated toward sport injury prevention. [↑](#footnote-ref-2)
3. Higher scores indicate students are more controlled motivated toward sport injury prevention. [↑](#footnote-ref-3)
4. Higher scores indicate students are more intended to engage in sport injury prevention in the forthcoming month. [↑](#footnote-ref-4)
5. Higher scores indicate students have more positive evaluation of sport injury prevention behaviours. [↑](#footnote-ref-5)
6. Higher scores indicate students have higher perceptions of the influence of significant others on the sport injury prevention behaviours. [↑](#footnote-ref-6)
7. Higher scores indicate students have higher perceived behavioural capability and controllability with respect to the sport injury prevention behaviours. [↑](#footnote-ref-7)
8. Higher scores indicate students spend more time and effort in sport injury prevention. [↑](#footnote-ref-8)
